# Monitoring of Pesticide Residues in Commonly Used Fruits and Vegetables in Kuwait

**DOI:** 10.3390/ijerph14080833

**Published:** 2017-07-25

**Authors:** Mustapha F. A. Jallow, Dawood G. Awadh, Mohammed S. Albaho, Vimala Y. Devi, Nisar Ahmad

**Affiliations:** Environment and Life Sciences Research Center, Kuwait Institute for Scientific Research, P.O. Box 24885, Safat 13109, Kuwait; dawadh@kisr.edu.kw (D.G.A.); mbahouh@kisr.edu.kw (M.S.A.); ydevi@kisr.edu.kw (V.Y.D.); nahmed@kisr.edu.kw (N.A.)

**Keywords:** pesticide residues, vegetables, fruits, food safety, GC-MS, LC-MS/MS, Kuwait

## Abstract

The presence of pesticide residues in primary and derived agricultural products raises serious health concerns for consumers. The aim of this study was to assess the level of pesticide residues in commonly consumed fruits and vegetables in Kuwait. A total of 150 samples of different fresh vegetables and fruits were analyzed for the presence of 34 pesticides using the quick easy cheap effective rugged and safe (QuEChERS) multi-residue extraction, followed by gas chromatography-mass spectrometry (GC-MS) or liquid chromatography-tandem mass spectrometry (LC**-**MS**/**MS). Pesticide residues above the maximum residue limits (MRL) were detected in 21% of the samples and 79% of the samples had no residues of the pesticides surveyed or contained residues below the MRL. Multiple residues were present in 40% of the samples with two to four pesticides, and four samples were contaminated with more than four pesticide residues. Of the pesticides investigated, 16 were detected, of which imidacloprid, deltamethrin, cypermethrin, malathion, acetamiprid, monocrotophos, chlorpyrifos-methyl, and diazinon exceeded their MRLs. Aldrin, an organochlorine pesticide, was detected in one apple sample, with residues below the MRL. The results indicate the occurrence of pesticide residues in commonly consumed fruits and vegetables in Kuwait, and pointed to an urgent need to develop comprehensive intervention measures to reduce the potential health risk to consumers. The need for the regular monitoring of pesticide residues and the sensitization of farmers to better pesticide safety practices, especially the need to adhere to recommended pre-harvest intervals is recommended.

## 1. Introduction

Pesticides are considered a vital component of modern farming, playing a major role in maintaning high agricultural productivity. Consequently, in high-input intensive agricultural production systems, the widespread use of pesticides to manage pests has emerged as a dominant feature [1]. However, reliance on pesticides is difficult to sustain because of unintended long-term adverse effects on the environment and human health in particular [2]. Pesticide residues are present in all agro-ecosystems, but the real risk to human health is through exposure to residues in primary and derived agricultural products [3]. Various human health related concerns are associated with pesticides, ranging from short-term impacts such as headaches and nausea, to chronic impacts, such as various cancers, birth defects, infertility, and endocrine disruption [4,5]. Children, in particular, are more endangered by short-term and chronic exposure to pesticides [6].

Like other countries aiming to facilitate self-sufficiency in food production, Kuwait has rapidly increased its agricultural pesticide use, especially on vegetable crops [7]. Most vegetables in Kuwait are produced under protected environments, accounting for more than 90% of the total greenhouse crop production [8]. With the intensive use of pesticides in greenhouses, crops grown under these protected environments may be prone to an increased level of pesticide residue than similar crops grown in the open field [9]. The annual consumption of pesticides in Kuwait was about 4.5 kg·ai.ha^−1^ per year in 2007 [10], and this has increased to 12.8 kg·ai.ha^−1^ per year by 2015 [7]. A total of 76 pesticide active ingredients, including pyrethroids, organophosphates and carbamates, were found to be in use, and 9% of these belong to the World Health Organization (WHO) toxicity class Ib (highly hazardous) [7]. Not only is this high input of pesticides perceived as necessary, but pesticide mixtures are also generally considered desirable. Farmers tend to apply pesticides too close to harvest because of lack of adequate knowledge regarding the safe and judicious use of pesticides [11], potentially contaminating the crop prior to sending their produce to the market. Pesticide residues have been detected in a number of vegetables and other food products in Kuwait [12,13,14,15,16,17]. Although some residue levels were below the maximum limits allowed, a few were above the limits established for these pesticides in food.

In order to ensure food safety for consumers and protect human health, many organizations and countries around the world have established maximum residue limits (MRLs) for pesticides in food commodities [18,19,20]. The MRL is the maximum level of a pesticide residue (expressed in mg·kg^−1^) which is legally permitted in or on food or animal feed [19]. Regulatory and enforcement mechanisms are also put in place by governments to monitor compliance of MRLs in food commodities. Although regulations on MRLs in food commodities exist in Kuwait using Codex Alimentarius Commission (Codex) MRLs as a reference point, these regulations are often not fully enforced. Because of the intensive use of pesticides in Kuwaiti agriculture, systemic investigations are necessary to verify the current status of pesticide residues in different agricultural produce. The objective of this study was to assess the levels of pesticide residues in commonly used fruits and vegetables in Kuwait for use as a reference for future monitoring. The information is also critically important for developing measures that aim at reducing or preventing human health risks from harmful pesticide residues in primary and derived agricultural products. The pesticides included in this study, except organochlorines, were selected on the basis of their wide use in vegetable production in Kuwait [7]. Most organochlorine pesticides, including DDT, have long been banned in Kuwait. However, their long-term persistence in the environment, especially in agricultural soils [21], and the possibility of entering the food chain via plant uptake [22] demand a continuous monitoring of these pesticides. Consequently, residues of organochlorine pesticides and their metabolites were also monitored in this study.

## 2. Materials and Methods

### 2.1. Chemicals and Reagents

Pesticide reference standards including malathion, diazinon, profenofos, primiphos-methy, chlorpyrifos-methyl, monocrotophos, dimethoate, cypermethrin, deltamethrin, fenpropathrin, oxamyl, imidacloprid, acetamiprid, metalaxyl, difenoconazole, α-BHC, β-BHC, γ-BHC, δ-BHC, heptachlor, aldrin, heptachlor epoxide, trans-chlordane, α-endosulfan, β-endosulfan, cis-chlordane trans-nonachlor, dieldrin, P-P-DDE, endrin, cis-nonachlor, P-P-DDD, endosulfan-sulfate, P,P-DDT, and methoxychlor were purchased from Dr. Ehrenstorfer GmbH (Augsburg, Germany), with certified purity ranging from 97% to 99%. Acetonitrile, tolune, ammonia solution, sodium chloride, acetic acid, triphenylphosphate (TPP), and ethoprophos were obtained from Merck (Darmstadt, Germany), and anhydrous magnesium sulfate, Bondesil sorbents (primary and secondary amine; PSA particle size 40 μm), and graphite carbon black (GCB) from Sigma-Aldrich (St. Louis, MO, USA). All the organic solvents used were higher performance liquid chromatography (HPLC) grade.

### 2.2. Sample Collection

A total of 150 fruit and vegetable samples were collected from September 2015 to August 2016 for pesticide residue analysis. The samples included eight pesticide-intensive vegetables (tomato, bell pepper, eggplant, cucumber, zucchini, cabbage, carrot, and potato) and four fruits (strawberry, watermelon, apple, and grapes), and are representative of commonly consumed commodities in Kuwait. The vegetable samples were collected from farmers’ fields in Kuwait’s two major agricultural regions (Wafra and Abdally). The fruit samples were purchased from a local market, and were originally from China (apple) and Jordan (strawberry, watermelon, and grapes). The sampling was performed in accordance with the general principles and methods of the European Commission (EC) directive 2002/63/EC [23] for establishing MRLs in food commodities. Each representative vegetable or fruit sample was a composite of 10 subsamples of the same commodity collected through random sampling. All the samples (1–2 kg each) were placed in sterile polythene bags, in an ice chess box, to avoid contamination and deterioration, labeled, and transported to the laboratory for processing. A representative portion (200 to 250 g) of the samples was chopped into small pieces and blended using a food processor. The homogenized samples were analyzed immediately or stored in stainless steel jars at 4 °C and analyzed within 24 h. Vegetable and fruit samples purchased from a local shop (Naureland, Kuwait) that deals with imported organic produce from Germany were utilized for the recovery studies and for the preparation of the matrix-matched calibration standards. The samples were analyzed and were shown to be free of pesticides.

### 2.3. Sample Extraction and Clean-Up

The extraction and clean-up method used was based on QuEChERS (quick easy cheap effective rugged and safe) sample preparation method for pesticides [24]. An aliquot of 15 g of homogenized sample was placed in a 50 mL centrifuge, and 15 mL of acetonitrile was added. The mixture was vortexed for one minute, followed by adding 4 g of magnesium sulphate and 1 g of sodium chloride. The sample was centrifuged, and the supernatant was removed for clean-up. The clean-up was carried out by transferring the supernatant into another tube containing 50 mg of primary and secondary amine (PSA), 50 mg of graphite carbon black (GCB) and 150 mg of magnesium sulphate. After agitation and centrifugation, the aliquots of the extract were reconstituted to 3 mL with acetonitrile for liquid chromatography-tandem mass spectrometry (LC-MS/MS) analysis and 1 mL with toluene for gas chromatography-mass spectrometry (GC-MS) analysis.

### 2.4. Solutions and Standards

Standard pesticide mix (1 mg·mL^−1^) stock solutions were prepared in toluene and acetonitrile for GC-MS and LC-MS/MS analysis, respectively. Multi-residue working solutions containing pesticides analyzed by GC-MS and LC-MS/MS were prepared in acetonitrile at a concentration of 5 ng·μL^−1^ and 0.5 ng·μL^−1^ for each pesticide, respectively. A 2% TPP solution in acetonitrile with 1% acetic acid was used as quality control (QC) standard for the GC-MS analysis. A 5 ng·μL^−1^ ethoprofos solution was prepared in acetonitrile to serve as internal standard (IS) for the LC-MS/MS determination, and in toluene as IS for the GC-MS determination.

### 2.5. Gas Chromatography-Mass Spectrometry (GC-MS) Analysis

A Shimadzu (QP 2010 GC-MS) gas chromatography equipped with mass selective detector and a RTX-5MS column (30 m long, 0.25 mm internal diameter, and 0.25 μm film thickness) was used for analysis. Sample injection was performed in the splitless mode, with an injector temperature of 250 °C and an interface temperature of 250 °C. The temperature of the oven was programed from an initial value of 90 °C for 2 min, ramped to 160 °C at 15 °C·min^−1^ for 10 min, and to 250 °C at 20 °C·min^−1^ for 15 min, and was raised to 270 °C at 20 °C·min^−1^ for 20 min. Helium was used as a carrier gas with a constant flow rate of 0.75 mL·min^−1^. Electron ionization was used at –70 eV in selective ion monitoring (SIM) and full-scan modes between 50 *m*/*z* and 500 *m*/*z* for the detection of different analytes. The following organochlorine pesticides and their metabolites were analyzed with GC-MS: α-BHC, β-BHC; γ-BHC; δ-BHC; heptachlor; Aldrin; heptachlor epoxide; trans-chlordane; α-endosulfan; β-endosulfan; cis-chlordane trans-nonachlor; dieldrin; P-P-DDE; endrin; cis-nonachlor; P-P-DDD; endosulfan-sulfate; P,P-DDT; methoxychlor. Similarly, the organophosphate pesticides (primiphos-methy, chlorpyrifos-methyl, monocrotophos, malathion, diazinon, profenofos, and dimethoate) and pyrethroid (cypermethrin, deltamethrin, and fenpropathrin) were analyzed with GC-MS.

### 2.6. Liquid Chromatography-Tandem Mass Spectrometry (LC**-**MS**/**MS) Analysis

LC-MS/MS analysis was performed using a liquid chromatography (Agilent 1200, Santa Clara, CA, USA) couple with a triple quadrupole mass detector (Agilent 6460), and an Agilent ZORBAX C-18 analytical column of 50 mm × 2.1 mm internal diameter and 1.8 μm particle size. The sheath gas temperature was kept at 400 °C, and the sheath gas flow was 12 L·min^−1^. Deionized water containing 0.1% formic acid (mobile phase A), and acetonitrile and deionized water (95:5, *v*/*v*) containing 0.1% formic acid (mobile phase B) were used for the gradient program, which started with 10% B for 3 min and was linearly increased to 90% B over 15 min. The column was then reconditioned for 20 min back to 10% B. The column temperature was kept at 35 °C, and the injection volume was 10 μL with a constant flow rate of 0.6 mL·min^−1^. For each compound, two multi reaction monitoring (MRM) transitions were monitored. The pesticides, oxamyl, imidacloprid, acetamiprid, metalaxyl, and difenoconazole, were analyzed with LC-MS/MS.

### 2.7. Quality Control

Calibration curves of each pesticide of interest were carried out in accordance with the European Commission guidelines [25]. Matrix-matched calibration standards were prepared in tomato blank acetonitrile extracts, using the multi-residue working solutions to reach a concentration ranging from 0.01 to 10 μg·L^−1^, and the IS (ethoprofos in acetonitrile) was added for LC-MS/MS determination or (ethoprofos in toluene) for GC-MS determination. Areas under the peak versus concentrations were fitted using linear regression to obtain the equation for the standard curves for the tested pesticides. Good linearity and reproducibility of calibration curves were achieved (r^2^ > 0.94). The performance of the QuEChERS method was evaluated by performing recovery studies [26,27]. The recovery rate and precision of the method (expressed as relative standard deviation (RSD), %) were measured by analyzing replicate pesticide-free samples of each type of vegetable or fruit, which were fortified at a concentration of 0.01 or 0.05 mg·kg^−1^ for each pesticide. Sensitivity was evaluated by determining the limit of detection (LOD) and limit of quantification (LOQ), using the signal-to-noise ratio (S/N) of 10:1 and 3:1, respectively. The recovery values ranged from 85% to 106% (precision range, 1.34% to 6.8%) for the 0.01 mg·kg**^−1^** concentration and 79% to 100% (precession range, 2.04% to 10.18%) for the 0.05 mg·kg^−1^ concentration (Table 1). The LOD for the pesticides ranged from 0.0007 to 0.214 mg·kg^−1^ and the LOQ from 0.0029 to 0.4521 mg·kg^−1^ (Table 1). All the pesticides LOD and LOQ values were lower than the MRLs established by Codex [19] for the fruits and vegetables sampled.

## 3. Results

### 3.1. Pesticide Residues in Analyzed Samples

The level of pesticide residues in 150 vegetable and fruit samples was determined. Pesticide residues were not detected in 62 samples (42%), while 88 samples (58%) contained detectable amount of pesticide residues (Table 2).

A total of 32 samples (21%) contained pesticide residue above the MRLs, whereas 56 samples (37%) contained pesticide residue at or below the MRLs established by Codex [19]. The percentage of contaminated samples was high for all of the vegetables except for carrot, zucchini, and cabbage. Tomato (88%), bell pepper (83%), and cucumber (87%) had the highest number of contaminated samples. No pesticide residues were detected in any of the potato samples. Only 17% of the zucchini samples, 10% of the carrot samples, and 7% of the cabbage samples were contaminated, and the residue levels in these vegetables were all below the MRLs set by Codex (Table 2). All of the fruit samples had a percentage of contaminated samples above 65%: strawberry (70%); grapes (86%); apples (90%), and; watermelon (83%). Tomato (19%), watermelon (50%), apple (80%), grapes (50%), and strawberry (40%) recorded the highest number of samples that exceeded the MRLs (Table 2).

### 3.2. MRL Exceedances and Detection Frequencies of Pesticides in Analyzed Samples

**Figure 1:**
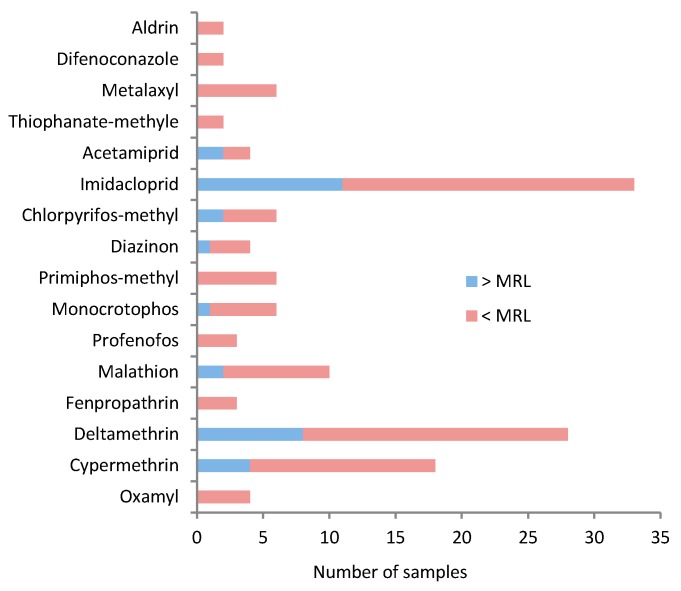
Type of pesticide detected and frequency of detection in fruit and vegetable samples. For each pesticide detected, the number of samples with residue below MRL or above MRL is indicated.

Of the 34 pesticides (including metabolites) studied, 16 pesticides were detected in the analyzed fruit and vegetable samples (Figure 1).

All detected active ingredients were insecticides (81%) and fungicides (19%). While 62% (8 out of 13 active ingredients) of the detected insecticides were found to exceed the MRL in at list one analyzed sample, none of the fungicides detected exceeded the MRL. The most common pesticides detected were deltamethrin (28 samples), imidacloprid (33 samples), cypermethrin (18 samples), malathion (10 samples), primiphos-methyl (six samples), chlorpyrifos-methyl (six samples), and metalaxyl (six samples) (Figure 1). Imidacloprid was detected in tomato, bell pepper, eggplant, cucumber, zucchini, watermelon, apple, grapes, and strawberry, but only tomato (two samples), cucumber (three samples), watermelon (one samples), and apple (five samples) exceeded the MRLs (Table 3). Two cucumber samples had considerable levels of imidacloprid (1.2 mg·kg^−1^). Residues of deltamethrin exceeding the MRLs were detected in watermelons (four samples), apples (one sample), and grapes (three samples). Residues of cypermethrin that exceeded the MRLs were also found in one sample of tomato, one sample of eggplant, and two samples of grapes. MRL exceedances were also detected for monocrotophos (one watermelon sample), acetamiprid (two strawberry samples), and chlorpyrifos-methyl (two grape samples) (Table 3). A residue of diazinon was found to exceed the MRL in one sample of strawberry. Residues of diazinon were also found in two apple samples but were below the MRL. Malathion was found in two apple samples, with detected residue levels exceeding the MRL. Only one organochlorine pesticide (aldrin) was detected in all of the samples analyzed. Residue of aldrin was detected in one apple sample, but was below the MRL (Table 3).

### 3.3. Multiple Residues in Analyzed Samples

**Figure 2:**
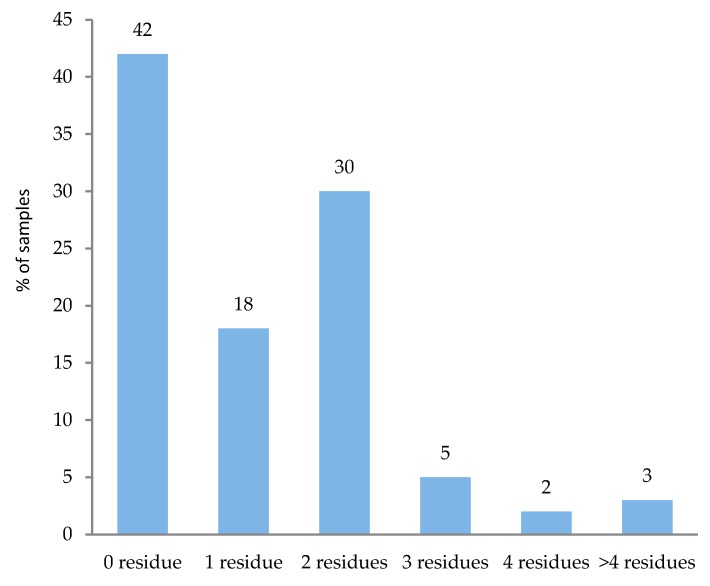
Fruit and vegetable samples with multiple residue of pesticides.

Fruits and vegetable samples containing no residue, one, and multiple residues are shown in Figure 2. Some samples contained only one pesticide residue, but 40% (60 out of 150) of the samples had multiple residues of more than one pesticide found in the same sample. A single residue was detected in 18% of the samples, and two, three, and four residues in 30%, 5%, and 2% of the samples, respectively. Few samples (3%) were contaminated with more than four pesticide residues. Multiple residues were found most frequently in tomato, cucumber, bell pepper, and watermelon. The most frequent combinations of two pesticides detected in the same sample were imidacloprid and deltamethrin (16 samples), and cypermethrin and deltamethrin (10 samples).

## 4. Discussion

This study shows the evidence of the presence of pesticide residues in fruits and vegetables in Kuwait. Slightly more than 20% of the samples analyzed contained pesticide residue above the MRL. Only potatoes were free from detectable residues. The findings of this study confirm previous studies that pointed to high levels of pesticide residues in fruit and vegetable commodities in Kuwait [15,16,17]. However, in comparison, the levels of the residues in the tested fruits and vegetables were below those previously reported in other studies [6,28,29]. Imidaclropid, a systemic neonicotinoid insecticide used to control various pests of fruits and vegetables in Kuwait [7], is the most commonly detected pesticide. Although imdaclropid is classified as moderately hazardous [30], this pesticide has been associated with human neurotoxicity [31]. Pesticides that are classified as highly hazardous (diazinon, oxamyl, and monocrotophos) [30] were also detected in some of the samples.

Among the organochlorine pesticide studied, only aldrin was detected in one sample of apple imported from China, but this residue was below the MRL for this crop. Because of their extreme harmful effects on human health and their long-term persistence in the environment [21], organochlorine pesticides are banned or restricted in majority of countries [32,33]. However, these pesticides are still being used clandestinely in some developing countries [34,35]. Previous studies have detected organochlorine pesticide residues in different commodities, including vegetables from Ghana, Saudi Arabia, and China [29,36,37], and fruits from China [38], but most residues were below the MRL allowed. Nevertheless, continuous consumption of food products even with moderate pesticide contamination may have negative consequences on human health in the long term [39]. Pesticides can accumulate in the tissues of organisms as they are not easily soluble [40].

The occurrence of multiple residues in some of the samples analyzed is likely to be a consequence of the application of different types of pesticides to protect a crop against different insect pests and diseases, especially vegetable crops in greenhouse environments where the incidence of pests can be extremely high [41]. The occurrence of multi-residue pesticide contamination in different commodities has also been reported in other investigations [9,36]. From the results, it is plausible to state that farmers were not following proper precautions with regard to the use of pesticides in appropriate dosages and at standard pre-harvest intervals. Consequently, a large number of fruit and vegetable samples were contaminated with pesticides. The high pesticide levels in some of the samples would suggest that these pesticides have been used indiscriminately, which could lead to health problems not only to the farmers but also to the general consumers. The widespread and overuse of pesticides in Kuwaiti agriculture, especially in greenhouse vegetable production, is a serious problem. Faced with several pest complexes, farmers simply rely on pesticides to address pest problems because of lack of viable alternative methods of pest control. Jallow et al. [7] reported that 58% of smallholder vegetable farmers in Kuwait overused pesticides. Pesticide application frequency in vegetable crops ranged from two times a month to once a week, depending on the crop [7]. This problem is further exacerbated by farmers’ limited knowledge of pesticide safety [11].

Greater priorities must be given to develop strategies for pesticide reduction in agriculture through farmer training in judicious and safe pesticide use, and promote alternatives to chemical pest control such as biological control. Intervention strategies by regulatory agencies to strengthen the enforcement mechanisms of current pesticide laws at the farm and retail level are a necessity in promoting safe pesticide use. Adherence to pesticide label instructions, especially pre-harvest intervals, needs to be ensured. It is also critical to raise awareness among the general public, who may be directly or indirectly exposed to pesticides, about the risk of these chemicals and how to reduce this risk. Consumers should be aware of practical measures to reduce the contamination of pesticides in fresh agricultural produce, especially fruits and vegetables that may be consumed raw. For example, washing, boiling, and especially peeling, have been demonstrated to reduce pesticide residues on fruits and vegetables [42,43]. Consequently, a follow-up investigation is needed to determine whether peeling, in particular, could reduce the dietary intake of pesticide residues in Kuwait. Finally, due to increasing trend in pesticide use in Kuwait, routine monitoring of pesticide residues in agricultural produce is a necessity to ensure the safety of consumers.

## 5. Conclusions

This study investigated the levels of pesticide residues in commonly used fruits and vegetables in Kuwait. The results indicated that majority of the fruit and vegetable samples were contaminated with pesticide residues, with concentrations above the MRL. From a public health perspective, the observed levels of pesticide residues pose a potential health risk to consumers. Therefore, to reduce this risk, sensitization of farmers to better pesticide safety practices and the need for continuous pesticide residue monitoring is highly recommended.

## Figures and Tables

**Table 1:** Determinations of pesticides in fruit and vegetable samples.

Pesticide	% Recovery (RSD) ^a^		LOD (mg·kg^−1^) ^b^	LOQ (mg·kg^−1^) ^c^
	Fortification Levels (mg·kg^−1^)			
	0.01	0.05		
Oxamyl	94 (4.32)	89 (8.45)	0.0020–0.0031	0.0059–0.0212
Cypermethrin	86 (5.46)	92 (6.42)	0.0013–0.0030	0.0051–0.0098
Deltamethrin	89 (3.21)	86 (4.98)	0.0019–0.0028	0.0034–0.0061
Fenpropathrin	102 (6.80)	94 (2.04)	0.0700–0.1869	0.2140–0.4521
Malathion	98 (2.67)	100 (3.98)	0.0040–0.0092	0.0162–0.0312
Profenofos	100 (5.47)	89 (4.21)	0.0018–0.0021	0.0031–0.0074
Monocrotophos	96 (5.21)	89 (6.21)	0.0012–0.0021	0.0069–0.0123
Primiphos-methyl	98 (4.89)	100 (10.18)	0.0026–0.0089	0.0163–0.0281
Diazinon	85 (1.34)	79 (4.51)	0.0018–0.0025	0.0096–0.0184
Chlorpyrifos-methyl	106 (4.82)	97 (8.23)	0.0016–0.0027	0.0057–0.0098
Imidacloprid	95 (6.21)	89 (6.18)	0.0009–0.0014	0.0036–0.0075
Acetamiprid	99 (3.21)	100 (2.98)	0.0021–0.0031	0.0098–0.0123
Thiophanate-methyl	102 (6.18)	87 (3.21)	0.0035–0.0192	0.0632–0.0971
Metalaxyl	92 (3.41)	85 (6.21)	0.0007–0.0021	0.0029–0.0036
Difenoconazole	93 (4.29)	90 (4.21)	0.0019–0.0034	0.0067–0.0132
Aldrin	89 (3.19)	85 (2.89)	0.0008–0.0012	0.0049–0.0086

^a^ Numbers in parenthesis represent relative standard deviation (RSD); ^b^ LOD: Limit of detection; ^c^ LOQ: Limit of quantification.

**Table 2:** Number of fruit and vegetable samples without pesticide residue, and with residue below the maximum residue limits (MRL) and above the MRL ^a^.

Produce	Number of Samples	Without Residue	With Residue < MRL	With Residue > MRL
Tomato	16	2 (12%)	11 (69%)	3 (19%)
Carrot	10	9 (90%)	1 (10%)	0 (0%)
Bell pepper	12	2 (17%)	10 (83%)	0 (0%)
Eggplant	14	5 (36%)	8 (57%)	1 (7%)
Cucumber	15	2 (13%)	10 (67%)	3 (20%)
Zucchini	12	10 (83%)	2 (17%)	0 (0%)
Watermelon	12	2 (17%)	4 (33%)	6 (50%)
Cabbage	15	14 (93%)	1 (7%)	0 (0%)
Potato	10	10 (100%)	0 (0%)	0 (0%)
Apple	10	1 (10%)	1 (10%)	8 (80%)
Grapes	14	2 (14%)	5 (36%)	7 (50%)
Strawberry	10	3 (30%)	3 (30%)	4 (40%)
Total	150	62 (42%)	56 (37%)	32 (21%)

^a^ Numbers in parenthesis represent percentages of samples without residue, with residue below MRL or above MRL from the total number of samples analyzed for each produce.

**Table 3:** Concentration ranges of pesticide residues in fruit and vegetable samples analyzed.

Pesticide	Tom	Car	Bpep	EgP	Cuc	Zuc	WaM	Cab	Pot	App	Grap	Straw
	Concentration Range (mg kg^−1^)											
Oxamyl	nd–0.09	nd	nd	nd	nd–0.98	nd	nd	nd	nd	nd	nd	nd
Cypermethrin	0.02–0.24 *	nd	nd	nd–0.13 *	nd	nd	nd–0.09	nd	nd	nd	nd–0.28 *	nd
Deltamethrin	nd	nd	nd–0.02	nd	nd	nd	0.06–0.29 *	nd	nd	0.2–0.32 *	0.032–0.38 *	nd–0.09
Fenpropathrin	nd	nd	nd	nd	nd	nd	nd	nd	nd	nd	nd	nd–1.2
Malathion	nd	nd	nd	nd	nd	nd	nd	nd	nd	0.09–0.58 *	nd	nd–0.98
Profenofos	0.02–0.39	nd	nd–0.03	nd	nd	nd	nd	nd	nd	nd	nd	nd
Monocrotophos	nd–0.02	nd	nd	nd	nd–0.04	nd	nd–0.02 *	nd	nd	nd	nd	nd
Primiphos–methyl	nd–0.1	nd	0.091–0.2	nd	nd	nd	nd	nd	nd	nd	nd	nd
Diazinon	nd	nd	nd	nd	nd	nd	nd	nd	nd	nd–0.08	nd	nd–0.12 *
Chlorpyrifos–methyl	nd–0.08	nd–0.03	nd–0.02	nd	nd	nd	nd	nd	nd	nd	nd–1.32 *	nd
Imidacloprid	nd–0.51 *	nd	nd–0.01	nd–0.09	0.05–1.2 *	nd–0.08	nd–0.23 *	nd	nd	0.2–0.65 *	nd–0.98	nd–0.2
Acetamiprid	nd	nd	nd–0.05	nd	nd	nd	nd	nd–0.1	nd	nd	nd	nd–1.01 *
Thiophanate–methyl	nd–0.3	nd	nd	nd	nb–0.19	nd	nd	nd	nd	nd	nd	nd
Metalaxyl	nd–0.2	nd	nd–0.01	nd	0.01–0.06	nd	nd–0.08	nd	nd	nd	nd	nd
Difenoconazole	nd	nd	nd	nd	nd	nd	0.04–0.1	nd	nd	nd	nd	nd
Aldrin	nd	nd	nd	nd	nd	nd	nd	nd	nd	nd–0.02	nd	nd

nd = pesticide residue not detected. * Pesticide residue above the MRL according to Codex [19]. Tom = Tomato; Car = Carrot; Bpep = Bell pepper; EgP = Eggplant; Cuc = Cucumber; Zuc = Zucchini; WaM = Watermelon; Cab = Cabbage; Pot = Potato; App = Apple; Grap = Grapes; Straw = Strawberry.
